# Spectrum of liver lesions hyperintense on hepatobiliary phase: an approach by clinical setting

**DOI:** 10.1186/s13244-020-00928-w

**Published:** 2021-01-12

**Authors:** Federica Vernuccio, Domenico Salvatore Gagliano, Roberto Cannella, Ahmed Ba-Ssalamah, An Tang, Giuseppe Brancatelli

**Affiliations:** 1 Department of Health Promotion, Mother and Child Care, Internal Medicine and Medical Specialties (PROMISE), University of Palermo, Via del Vespro, 129, 90127 Palermo, Italy; 2 University Paris Diderot, Sorbonne Paris Cité, Paris, France; 3 I.R.C.C.S. Centro Neurolesi Bonino Pulejo, Contrada Casazza, SS113, 98124 Messina, Italy; 4 Department of Biomedicine, Neuroscience and Advanced Diagnostics (Bi.N.D.), University Hospital of Palermo, Via del Vespro 129, 90127 Palermo, Italy; 5 Department of Biomedical Imaging and Image-Guided Therapy, Medical University of Vienna, General Hospital of Vienna (AKH), Waehringer Guertel 18-20, 1090 Vienna, Austria; 6 Department of Radiology, Centre Hospitalier de l’Université de Montréal (CHUM), Montreal, QC Canada; 7 Centre de Recherche du Centre hospitalier de l’Université de Montréal (CRCHUM), Montreal, QC Canada; 8 Department of Radiology, Radio-Oncology and Nuclear Medicine, University of Montreal, Montreal, Canada

**Keywords:** Liver neoplasms, Magnetic resonance imaging, Hepatocellular carcinoma, Gadoxetate disodium, Gadobenate dimeglumine

## Abstract

Hepatobiliary MRI contrast agents are increasingly being used for liver imaging. In clinical practice, most focal liver lesions do not uptake hepatobiliary contrast agents. Less commonly, hepatic lesions may show variable signal characteristics on hepatobiliary phase. This pictorial essay reviews a broad spectrum of benign and malignant focal hepatic observations that may show hyperintensity on hepatobiliary phase in various clinical settings. In non-cirrhotic patients, focal hepatic observations that show hyperintensity in the hepatobiliary phase are usually benign and typically include focal nodular hyperplasia. In patients with primary or secondary vascular disorders, focal nodular hyperplasia-like lesions arise as a local hyperplastic response to vascular alterations and tend to be iso- or hyperintense in the hepatobiliary phase. In oncologic patients, metastases and cholangiocarcinoma are hypointense lesions in the hepatobiliary phase; however, occasionally they may show a diffuse, central and inhomogeneous hepatobiliary paradoxical uptake with peripheral rim hypointensity. Post-chemotherapy focal nodular hyperplasia-like lesions may be tricky, and their typical hyperintense rim in the hepatobiliary phase is very helpful for the differential diagnosis with metastases. In cirrhotic patients, hepatocellular carcinoma may occasionally appear hyperintense on hepatobiliary phase.

## Key points

- In non-cirrhotic patients, FNH and FNH-like lesions are likely the most common lesions showing hyperintensity in the HBP.
- Cholangiocarcinoma and some metastases may demonstrate central contrast retention in the HBP due to fibrotic stroma.
- In cirrhotic patients, well-differentiated HCC may show contrast uptake in the HBP in 9–14% of the cases.

## Background

Hepatobiliary MRI contrast agents—i.e., gadobenate dimeglumine (i.e., Gd-BOPTA, Multihance, Bracco, Milan, Italy) and gadoxetate disodium (i.e., Gd-EOB-DTPA, Eovist or Primovist, Bayer Healthcare Pharmaceuticals, Whippany, NJ, USA)—are increasingly being used for liver imaging. These contrast agents are taken up by normal hepatocytes through the organic anion transporting polypeptide 1 (OATP1B3) on the sinusoidal surface and excreted into bile by multidrug-resistance-associated proteins (MRP2) on the canalicular surface [1]. Compared to gadobenate dimeglumine, gadoxetate disodium is administered at a lower dose (0.1 mmol/kg vs 0.025 mmol/kg of body weights), has greater uptake (50% vs. 3–5%) and has earlier onset uptake by the hepatocytes (starting from 40 min vs. 60–90 s after contrast injection), which results in differences in the enhancement of hepatic parenchyma and vessels on portal venous, delayed and hepatobiliary phase (HBP), as well as earlier acquisition of HBP [2–6].

**Fig. 1:**
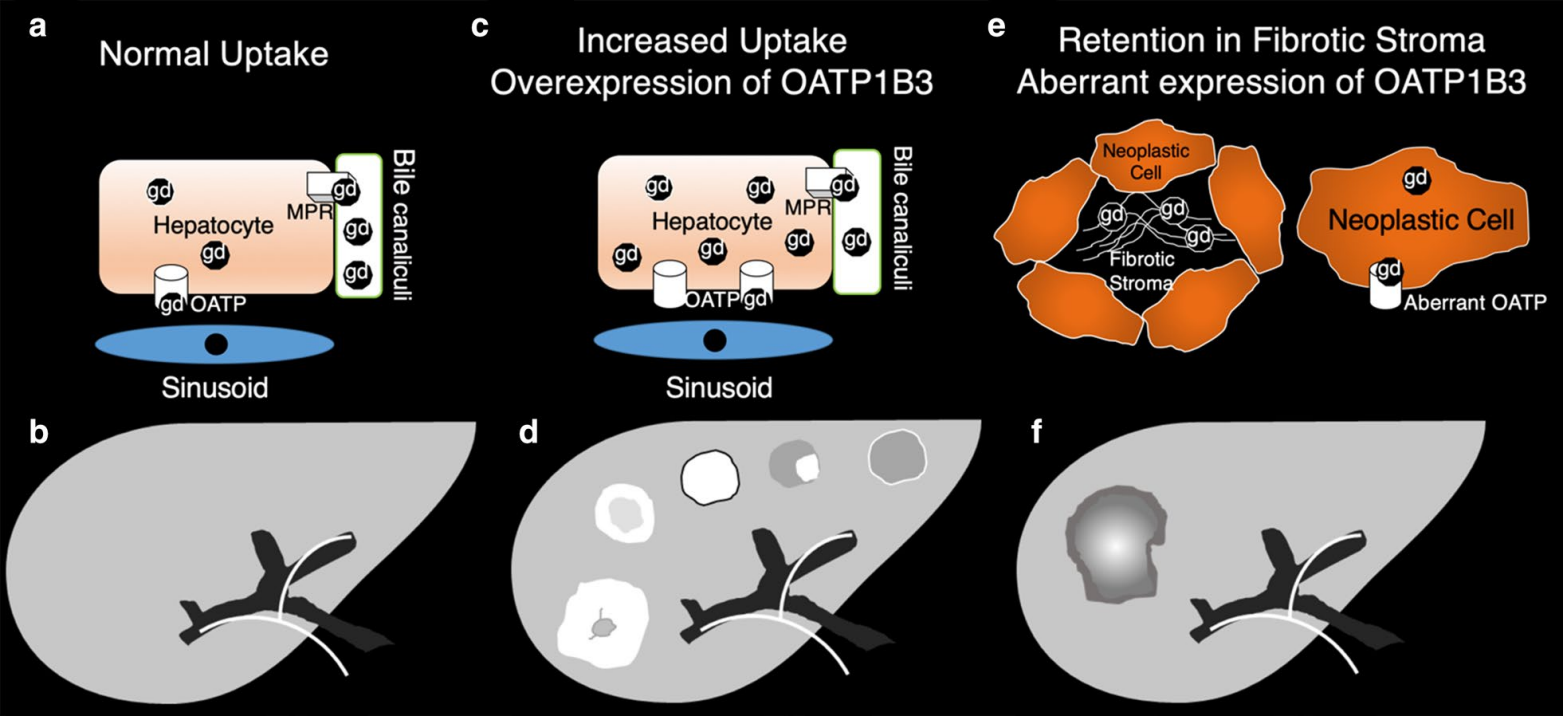
Top row: schematics showing the uptake mechanism of hepatobiliary contrast agents. Bottom row: corresponding schematics of the signal characteristics of liver and focal observations. Left column: **a** uptake in hepatocytes and (**b**) corresponding signal characteristics in normal liver. Middle column: **c** increased uptake due to overexpression of OATP1B3 and (**d**) corresponding iso- or hyperintense signal characteristics of focal observations (from left to right: FNH, nodule with hyperintense rim, hyperintense HCC, HCC with nodule-in-nodule architecture and HCC with peritumoral hyperintensity). Right column: **e** increased uptake due to retention in fibrotic stroma or aberrant expression of OATP1B3 and (**f**) representative target imaging appearance (lower row) of intrahepatic cholangiocarcinoma or metastasis

The lack of normal hepatocytes in most focal liver lesions results in the lack of hepatobiliary contrast uptake and, therefore, hypointensity of these lesions relatively to normal background liver parenchyma in the HBP. Less commonly, hepatic lesions may show variable signal characteristics (Table 1) on HBP due to increased uptake of hepatobiliary contrast agents through OATP1B3 or to a delayed central enhancement secondary to retained contrast material by the fibrotic stroma (Fig. 1) [7, 8].

**Table 1:** Observations, mechanism, typical imaging features and prevalence of iso- or hyperintensity signal characteristics on hepatobiliary phase

Observations	Mechanisms	Typical imaging features	Prevalence of iso- or hyperintensity
*Non-cirrhotic patients*			
Focal nodular hyperplasia	Overexpression of OATP1B3; suggested also an increase in well-differentiated bile ducts	Iso- or hyperintensity; hyperintense rim on HBP	97%
Hepatocellular adenomas	Overexpression of OATP1B3	Iso- or hyperintensity	83%, 19%, 0% and 0% of β-catenin, inflammatory, HNF1α inactivated, and unclassified HCAs, respectively
Fat sparing in steatotic liver	Preserved or even increased parenchymal function compared to the background steatotic liver	Homogeneous iso- or hyperintensity	Nearly always (no specific data available)
*Primary or secondary vascular disorders*			
FNH-like nodule	Equal or overexpression of OATP1B3	Iso- or hyperintensity; hyperintense rim on HBP	100%
*Oncologic patients*			
FNH-like nodule	Equal or overexpression of OATP1B3	Iso- or hyperintensity; hyperintense rim on HBP	100%
Metastases	Retention in fibrotic stroma; aberrant expression of OATP1B3	Targetoid appearance	No specific data available
Cholangiocarcinoma	Retention in fibrotic stroma	Targetoid enhancement	42–57%
*Cirrhotic patients*			
Hepatocellular Carcinoma	Overexpression of OATP1B3	Homogeneous hyperintensity, mosaic pattern, nodule-in-nodule appearance or peritumoral hyperintensity	8.8–14%
Regenerative or low-grade dysplastic nodules	Overexpression of OATP1B3	Homogeneous iso- or hyperintensity	No specific data available
Multiacinar regenerative nodules	Overexpression of OATP1B3	Hyperintense rim on HBP	No specific data available
Periportal hyperintensity	Possible regenerative changes of periportal hepatocytes	Bandlike hyperintense areas along the portal tracts	3%

This pictorial essay reviews a broad spectrum of benign and malignant focal hepatic observations that may show hyperintense signal intensity on HBP on MRI in non-cirrhotic patients, in patients with vascular disorders, in oncologic and cirrhotic patients.

## Non-cirrhotic patients

An incidental liver observation detected at imaging in an asymptomatic patient without underlying disease is benign in 96% of the cases [9]. Among these observations, those that may show iso- or hyperintensity in the HBP are mainly focal nodular hyperplasia (FNH), areas of fat sparing in steatotic liver, and, seldom, hepatocellular adenomas (HCAs). The likelihood of these observations depends on the patient's on age, gender and risk factors such as oral contraceptives, steroids, history of glycogenosis [10–17]. Cholangiocarcinoma and metastases may show some central enhancement in the HBP phase that is, however, typically lower in comparison with the background parenchyma [18–23].

### Focal nodular hyperplasia

FNH is the second most common benign liver tumor with a prevalence of 0.03–0.9% in the general adult population, with a peak incidence among women between 30 and 40 years old [24, 25]. FNH is defined as a nodule composed of benign-appearing hepatocytes occurring in a liver that is otherwise histologically normal or nearly normal [26]. Although FNH may increase in size in 3–15% of cases, these lesions do not evolve to malignancy and their management is conservative [27, 28].

**Fig. 2:**
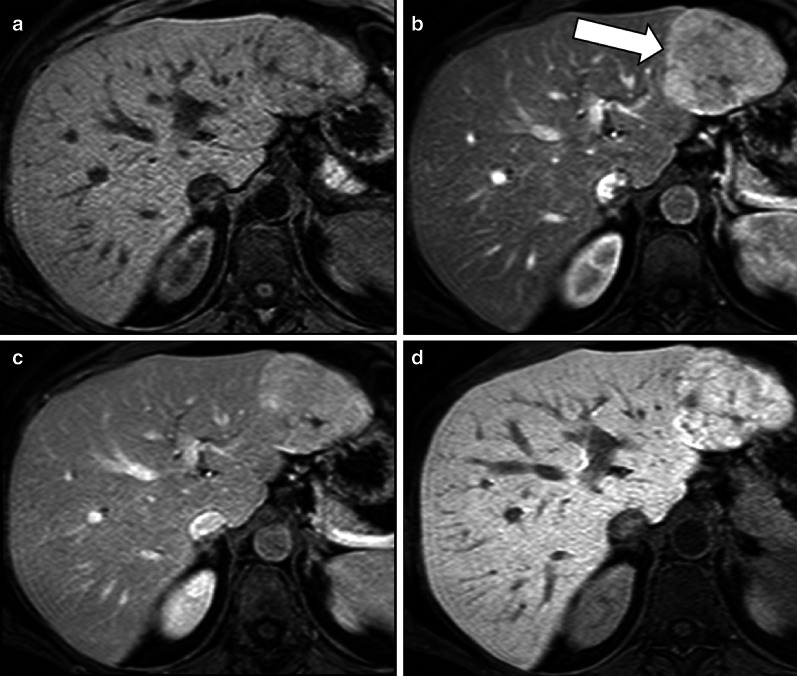
A 46-year-old woman with focal nodular hyperplasia. Gadoxetate disodium-enhanced MRI shows a focal nodular hyperplasia that is (**a**) nearly isointense to liver parenchyma in the precontrast T1-weighted sequence, (**b**) with marked hyperenhancement (arrow) in the arterial phase, (**c**) mildly hyperintense in the portal venous phase and (**d**) hyperintense in the hepatobiliary phase

FNHs show iso- or hyperintensity in the HBP relatively to liver parenchyma in the vast majority (97%) of cases [10] (Fig. 2), and this is attributed to OATP1B3 expression equal or higher than that of the background liver or to an increase in well-differentiated bile ducts in these lesions compared to the surrounding parenchyma [29–33].

This typical iso- or hyperintensity of FNH relatively to liver parenchyma in the HBP allows the differential diagnosis between FNH and HCA—which is hypointense relatively to liver parenchyma most of the time—with a specificity of 91–100% [10, 11, 34] and a superior accuracy compared to other morphological and dynamic vascular criteria alone and in combination [35]; in clinical practice, its presence decreases the number of indeterminate or inconclusive cases that require biopsy or surgery. Iso- or hyperintensity in the HBP is homogenous in 23–59% of cases [33, 36, 37]. The great variability of these percentages in the literature may be partially attributed to the subjective identification of different patterns of FNHs in the various studies. For instance, An et al. [36] identified seven different patterns of signal intensity in the HBP; van Kessel [33] used a six-point scale to describe FNH intensity as compared to surrounding liver parenchyma; Mohajer et al. [37] classified all FNHs in only 3 patterns (i.e., uniform uptake, iso- or hyperintense to liver, hyperintense rim with core that is hypointense relative to liver, or hyperintense rim with core that is iso- or hyperintense to liver) while a more recent paper identified two patterns for FNH in the HBP, including an homogenous or a doughnut-like pattern [38].

**Fig. 3:**
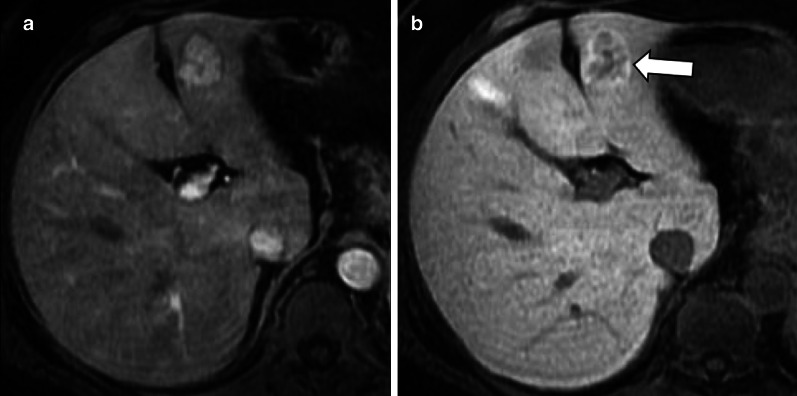
A 44-year-old woman with focal nodular hyperplasia. Gadobenate dimeglumine-enhanced MRI shows a focal nodular hyperplasia that is (**a**) hypervascular in the arterial phase and (**b**) demonstrates a hyperintense rim (arrow) in the hepatobiliary phase

A hyperintense rim on HBP with a peripheral hyperintensity higher than a central iso- or hypointense area is demonstrated in 23–66% of cases (Fig. 3) [29–33, 37]. The hyperintense rim on HBP is related at pathology to a strong OATP1B3 expression of the hepatocytes in the peripheral areas of the lesion, whereas the hepatocytes in the central areas, surrounding the thin radial scars, do not show such expression [30]. Although the causative mechanism of this different OATP1B3 expression is not fully understood, some theories have been proposed: Ven Kessel et al. [33] showed that FNH with hyperintense rim on HBP had fibrous tissue in the lesion center surrounded by some inflammation and vascular proliferation with ductular metaplasia, while the lesion periphery consisted mainly of well-differentiated preexistent bile ducts without signs of metaplasia, fibrous tissue, or inflammation; according to another theory, the reason for this different expression could be secondary to a different origin of the hepatocytes, with the ones surrounding the central scar of FNH originating from periportal venous hepatocytes and the ones in the peripheral portion from perivenular hepatocytes [29].

### Hepatocellular adenoma

HCA is an uncommon benign neoplasm more frequently detected in young women with history of oral contraceptive assumption [39, 40] or young men with history of anabolic steroids and glycogen storage disease and recently more increasing in both gender suffering from metabolic syndrome [12, 41]. HCAs warrant close follow-up and surgery in selected cases considering the possibility of progressive disease [42] and complications (i.e., bleeding) for those exceeding 5 cm in diameter despite treatment and, therefore, suspected of malignant transformation [27].

Iso- or hyperintensity of HCAs on HBP has been reported in a variable percentage, ranging from 0 to 70% [6], and this variability is primarily related to the fact that HCAs include eight different subtypes showing different molecular/genetic background [43]. Hepatocellular adenomas are divided into four main subgroups, showing specific immunohistochemical phenotype, molecular background, imaging findings, clinical settings and natural history: HNF1α-inactivated HCA, inflammatory HCA, β-catenin activated HCA, and argininosuccinate synthase 1-positive/sonic hedgehog HCA [43]. HCA without classical steatosis, mixed β-catenin-activated and inflammatory HCA and HCA with focal transformation into hepatocellular carcinoma (HCA–HCC) are also described [43]. Mixed β-catenin-activated and inflammatory and β-catenin-activated forms have the highest risk of malignant transformation due to their β-catenin (CTNBB1) exon 3 gene mutation, with a reported odds ratio more than 9 [43]. Hepatobiliary contrast agent retention in the HBP occurs in 83%, 19%, 0% and 0% of β-catenin, inflammatory, HNF1α-inactivated and unclassified HCAs, respectively [44], and it is helpful to distinguish all higher-risk HCA and HCA–HCC with 100% accuracy [45]. Activation of β-catenin protein causes uncontrolled hepatocyte proliferation and overexpression of OATP1B3 responsible for iso- or hyperintensity on HBP [32, 45, 46].

At pathology, OATP1B3 expression is preserved or increased not only in β-catenin–activated HCAs, but also in β-catenin–activated-inflammatory HCA and HCA–HCC; this latter shows also an increased MRP3 expression [45]. This information is of translational interest as the hyperintensity on HBP of HCAs could potentially be helpful in identifying HCAs at high risk of malignancy. Specifically, β-catenin–activated HCAs, β-catenin–activated-inflammatory HCA and HCA–HCC are expected to show a hyperintense signal on HBP, and HCA–HCC might show a faster sinusoidal excretion because of their increased MRP3 expression [44, 46].

**Fig. 4:**
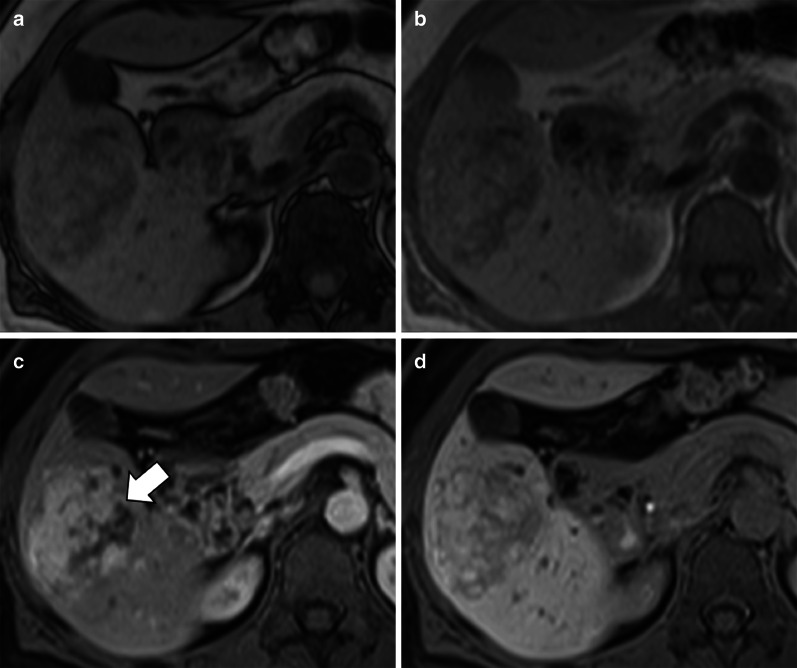
A 46-year-old woman with clinical history of oral contraceptive use and pathology-proven hepatocellular adenoma. Gadoxetate disodium-enhanced MRI shows a normal liver characterized by (**a**) no significant signal drop of hepatic parenchyma in the opposed phase compared to (**b**) the in-phase and (**c**) a hepatocellular adenoma (arrow) that shows contrast enhancement in the arterial phase and (**d**) heterogeneous hyperintensity in the hepatobiliary phase

An interesting finding of some studies is the relatively high percentage (21–67%) of inflammatory HCAs showing iso-hyperintensity on HBP (Fig. 4), which is in contradiction to the molecular background of these lesions [13–16, 32, 46, 47]. However, a possible explanation is the presence of marked hepatic steatosis that reduces signal intensity of background liver on T1-weighted pre- and post-contrast images—including the HBP—and modifies the relative signal intensity of HCAs [32]. Another possible reason could be that some HCAs included in these studies were in fact mixed β-catenin activated and inflammatory HCA.

### Fat sparing in steatotic liver

**Fig. 5:**
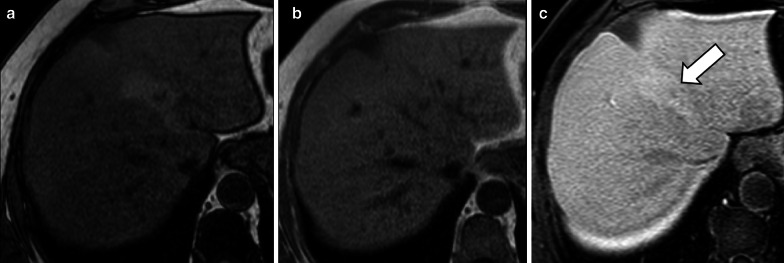
A 63-year-old man with liver steatosis and focal fat sparing area in segment 4. Gadoxetate disodium-enhanced MRI shows (**a**) in the opposed-phase and (**b**) in-phase images a fat sparing area with similar signal unlike the background liver that has marked signal drop in the opposed-phase sequence. The area of fat sparing is (**c**) slightly hyperintense (arrow) to the background liver in the hepatobiliary phase

Focal fatty sparing is a common finding in patients with diffuse fatty infiltration of the liver [48]. Areas of fat sparing in diffuse fatty infiltration are usually located in segment 2, caudate lobe, adjacent to gallbladder or may surround a liver lesion, and may present in different shapes (e.g., geographic, wedge-shaped, nodular) [48]. Fat sparing can be recognized on MRI as an area devoid of signal drop in the opposed-phase image compared to the in-phase. These areas are usually not visible on T2-, T1- and diffusion-weighted images and on post-contrast phases and may appear hyperintense in the HBP (Fig. 5) due to preserved or even increased parenchymal function [17]. The causative mechanism of focal fatty sparing is usually related to abnormal vascular inflow, due to aberrant small veins, arterial perfusion abnormalities or reduced portal flow and increased arterial flow in case of fatty sparing surrounding focal liver lesions [48].

## Patients with vascular liver disorders

Vascular liver disorders—e.g., Budd–Chiari syndrome, congenital portosystemic shunts, hereditary hemorrhagic telangiectasia, cavernous transformation of the portal vein—are associated with the development of hepatocellular tumors such as FNH-like nodules (more commonly), HCAs and HCC [49–52]. The causative mechanisms of hepatocellular lesions in vascular liver disorders include all causes of reduced portal venous inflow that consequently lead to an increased hepatic arterial inflow.

### FNH-like nodules

FNH-like nodules arise as a local hyperplastic response to vascular alterations, occurring in about 36% of patients with Budd–Chiari syndrome [49, 53].

FNH-like nodules may rarely be observed in patients with cirrhosis [54, 55] or can occur de novo after treatment with oxaliplatin, usually after a mean interval of about 48 months after treatment and may increase in size in 42% of the cases [56]. The prevalence of FNH-like nodules after treatment with oxaliplatin is not known [56, 57].

FNH-like lesions demonstrate enhancement in the arterial phase and persistent enhancement on portal venous or delayed phase in most cases; however, washout may be occasionally detected and, in these cases, the differential diagnosis with HCC is tricky [49, 58].

**Fig. 6:**
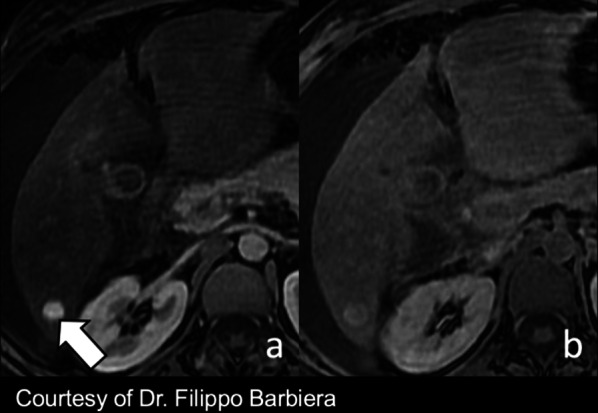
A 38-year old woman with Budd–Chiari syndrome and FNH-like nodule. Gadobenate dimeglumine-enhanced MRI demonstrates a FNH-like nodule that shows (**a**) arterial phase hyperenhancement (arrow) and (**b**) hyperintensity in the hepatobiliary phase

**Fig. 7:**
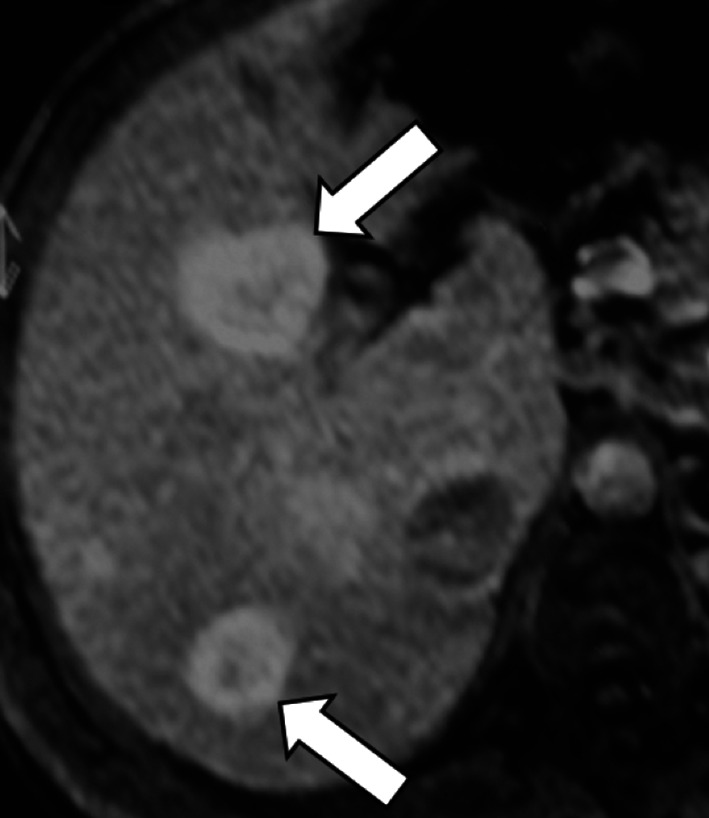
A 41-year-old man with cavernous transformation of the portal vein and FNH-like nodules. Gadoxetate disodium-enhanced MRI shows two FNH-like nodules (arrows) that are hyperintense in the hepatobiliary phase with central small hypointensity due to a central scar

FNH-like lesions are usually iso- to hyperintense on HBP [49] due to equal or higher OATP1B3 expression compared with the background liver tissue [7]. In our experience, the signal intensity is homogenously iso- to hyperintense (Fig. 6) on HBP when lesions are small, while it may demonstrate a hyperintense rim—i.e., increased uptake in the periphery of the nodule and a central hypointense area (Fig. 7)—if the lesion is larger. Of note, Mamone et al. [59] have recently described the possibility of FNH-like lesions showing hypointensity on HBP and suggested as a potential explanation, either a different OATP1B3 expression in hepatocytes or the presence of areas of abnormal hepatic perfusion/congestion.

**Fig. 8:**
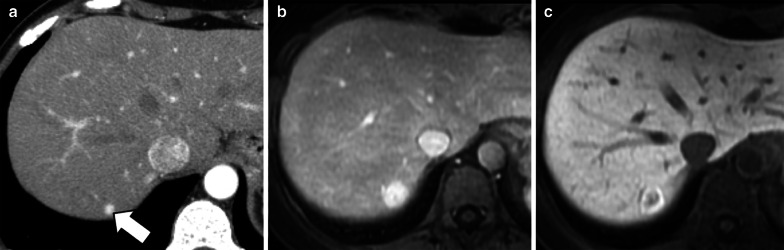
A 40-year-old woman with sigmoid adenocarcinoma and liver metastases treated with chemotherapy (XELOX regimen). **a** Contrast-enhanced CT shows a FNH-like nodule (arrow) that is hypervascular in the arterial phase. Gadoxetate disodium-enhanced MRI performed 2 years later (**b**) in the arterial phase and (**c**) hepatobiliary phase shows size increase which raises the suspicion of metastasis. However, the hepatobiliary phase (**c**) demonstrates a hyperintense rim establishing the diagnosis of FNH-like nodule

In case of FNH-like nodules related to oxaliplatin, FNH-like nodules are also usually hyper- or isointense to the surrounding liver parenchyma in the HBP, and a ring (or doughnut-like) pattern on HBP is observed in approximately 50% cases (Fig. 8) [56]. The potential explanation of the ring pattern on HBP is suggested to be similar to that described for FNH-like nodules in vascular liver disorders because the causative mechanism of FNH-like nodules after treatment with oxaliplatin is considered sinusoidal obstruction syndrome [56, 57]. FNH-like nodules do not have any risk of malignant transformation and, therefore, do not require any follow-up or treatment. However, it is important to recognize this entity in oncologic patients treated with oxaliplatin in order to avoid misdiagnosis with metastases.

Considering the challenges in the differential diagnosis between FNH-like lesions and HCC, specific diagnosis requires extensive clinical, laboratory and imaging work-up, including follow-up every 6 months if liver lesions have features of FNH-like lesions and alpha 1-fetoprotein levels are low, or liver biopsy if imaging features are atypical, if significant changes occur over time or if serum alpha 1-fetoprotein becomes elevated [49].

## Oncologic patients

**Fig. 9:**
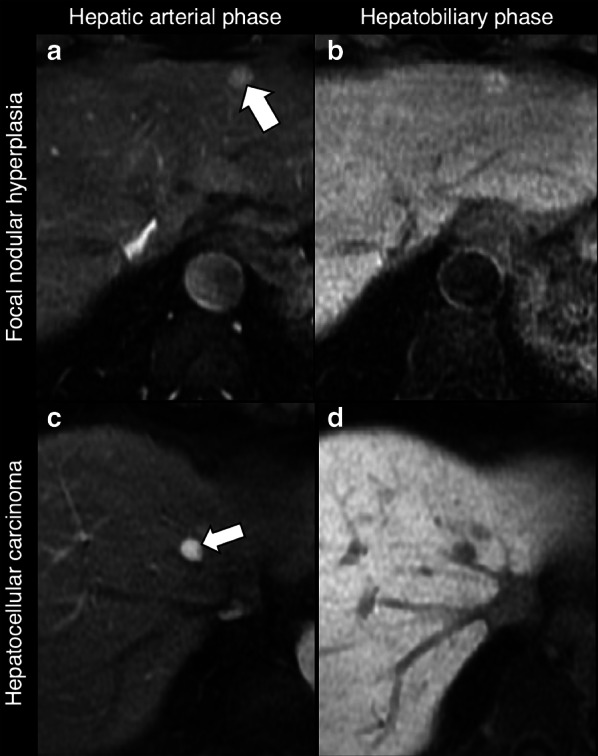
Differential diagnosis of hypervascular lesions in the arterial phase in oncologic patients. Top row: 53-year-old woman with breast cancer and focal nodular hyperplasia. Gadobenate dimeglumine-enhanced MRI demonstrates a focal nodular hyperplasia (arrow) that (**a**) enhances in the hepatic arterial phase and (**b**) is hyperintense in the hepatobiliary phase. Bottom row: 58-year-old man with pharyngeal carcinoma and hepatocellular carcinoma. Gadoxetate disodium-enhanced MRI demonstrates a small HCC nodule (arrow) that (**c**) enhances in the hepatic arterial phase and (**d**) is hypointense in the hepatobiliary phase

Oncologic patients are more likely to have metastases as compared to the general population, but benign observations showing HBP hyperintensity (e.g., FNHs) are expected to have the same incidence as in the general population [60–63]. Hypervascular benign liver lesions may simulate metastases and HBP often allows the differential diagnosis (Fig. 9). Iso-hyperintensity on HBP in a lesion detected in oncologic patients usually indicates benignity.

As discussed above, oncologic patients may show FNH-like nodules after chemotherapy, and the diagnosis of these lesions benefits from the use of hepatobiliary contrast agents.

In oncologic patients, malignant focal liver lesions showing variable signal characteristic on HBP include metastases (i.e., the most common malignant liver tumors overall) and intrahepatic cholangiocarcinomas (i.e., the most common primary non-hepatocellular carcinoma malignancy in non-cirrhotic liver).

### Metastases

Metastases are the most common malignant liver tumors [64]. Liver metastases usually originate from primary tumor of colon, breast, lung, pancreas or stomach. Liver metastases are broadly classified as hypoenhancing and hyperenhancing relative to the liver parenchyma on hepatic arterial phase. Most liver metastases are hypoenhancing and adenocarcinoma from the gastrointestinal tract is the most frequent source of these metastases [65]. Hyperenhancing metastases typically originate from primary neuroendocrine tumors, renal cell carcinoma, thyroid carcinoma, choriocarcinoma, and sarcomas. Metastases must be differentiated from other benign or malignant liver lesions that may occur in these patients.

The use of hepatobiliary contrast agents is particularly important in the evaluation of liver metastases because it increases sensitivity for detection of metastases compared to CT or extracellular agents providing high tumor-to-lesion contrast on HBP [66, 67]. The combination of MR with hepatobiliary contrast agents and diffusion-weighted imaging yields better diagnostic accuracy and sensitivity in the detection of small liver metastasis than each MR scan sequence alone, on both per-lesion basis and per-patient basis [68].

**Fig. 10:**
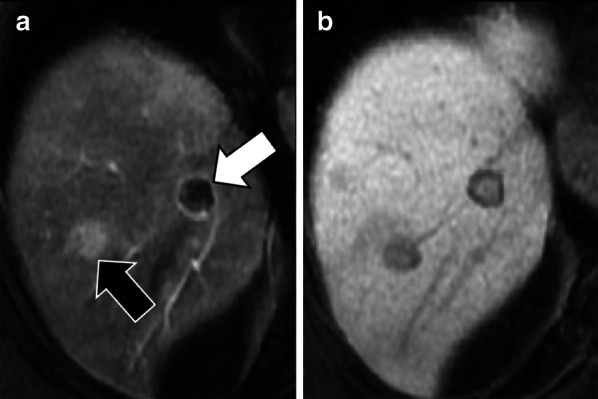
A 73-year-old man with colon cancer and liver metastases. Gadobenate dimeglumine-enhanced MRI in (**a**) arterial phase and (**b**) hepatobiliary phase demonstrates two liver metastases with different signal characteristics. A metastasis (black arrow) shows homogenous arterial phase hyperenhancement with a peripheral hypointense rim in the hepatobiliary phase. A second metastasis (white arrow) shows peripheral rim enhancement with a peripheral hypointense rim and cloud-like central enhancement in the hepatobiliary phase

Liver metastases are hypointense on HBP due to their lack of normal hepatocytes. However, metastases may occasionally demonstrate in the HBP central areas of relative hyperintensity—described as “EOB-cloud enhancement” similarly to cholangiocarcinoma—compared to surrounding lesion (rim) hypointensity with a resulting target appearance (Fig. 10) [22, 23, 67]. The causative mechanism of this phenomenon is still debated; it has been suggested to be a slow accumulation of the contrast material within the intercellular matrix of the tumor [22] or an interstitial diffusion of the hepatobiliary contrast agent within areas of necrosis [67]. Another possible theory is the presence of aberrant expression of OATP1B3 in liver metastases as possible explanation of the hepatobiliary uptake; however, while Park et al. [69] showed an increased expression of aberrant OATP1B3 (i.e., the protein involved in hepatocyte contrast uptake), Wlcek et al. [70] showed that expression of OATP1B3 is downregulated while other OATPs are upregulated. The target appearance of metastases in the HBP (i.e., peripheral hypointense rim compared to central cloud of enhancement) resembles the peripheral washout pattern occurring in the delayed extracellular phases in 24% of metastases [71].

### Cholangiocarcinoma

Intrahepatic cholangiocarcinoma is the most common primary non-HCC malignancy in non-cirrhotic liver [72]. Although the typical pattern of intrahepatic cholangiocarcinoma on dynamic studies (i.e., irregular peripheral enhancement in the hepatic arterial phase and gradual centripetal enhancement on following phases) usually allows a confident diagnosis, HBP images are useful to increase lesion conspicuity and better delineate daughter nodules and intrahepatic metastasis [73].

**Fig. 11:**
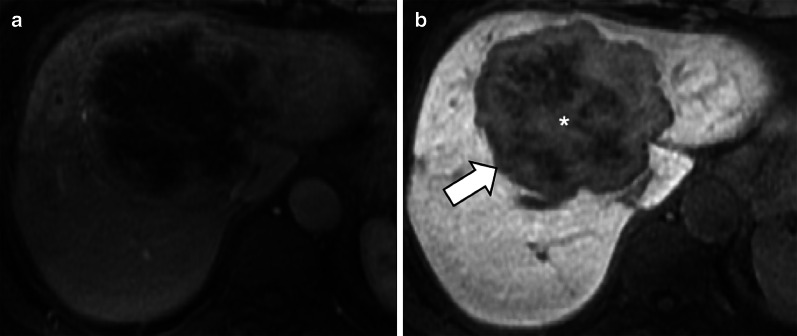
A 71-year-old woman with cholangiocarcinoma. Gadoxetate disodium-enhanced MRI shows an intrahepatic mass-forming cholangiocarcinoma with (**a**) continuous rim enhancement on hepatic arterial phase and (**b**) a target pattern on hepatobiliary phase with peripheral hypointense rim (arrow) and inhomogeneous contrast media uptake with a central enhancing area (asterisk) likely related to fibrous stroma

Intrahepatic mass-forming cholangiocarcinomas are hypointense on HBP because these lesions lack hepatocytes. In 42–57% of these tumors, a target sign is demonstrated as a peripheral hypointense rim and a diffuse, mainly central and inhomogeneous “EOB-cloud enhancement” (Fig. 11) [18–20].

The clinical relevance of the presence of iso- to hyperintense areas on HBP within cholangiocarcinomas is twofold: first it is helpful for the differential diagnosis with scirrhous HCC because scirrhous HCC is typically hypointense on HBP [74]; second, it correlates with prognosis, with a significantly lower rate of 5-year survival compared to those showing hypointensity on HBP (53% vs 87%, respectively; *p* = 0.048) [19].

## Cirrhotic patients

Cirrhotic patients require periodic surveillance for HCC screening. Decreased expression of OATP1B3 is one of the steps of hepatocarcinogenesis and leads to HBP hypointensity [75, 76]. Hyperintensity on HBP in a cirrhotic liver usually indicates benignity, but well-differentiated HCC in cirrhotic patients may also show hyperintensity on HBP. Although cholangiocarcinoma—i.e., the second most common primary hepatic malignancy—may show the so-called “EOB-cloud enhancement” on HBP, this pattern is uncommon in cirrhosis [77], and we hypothesize that this is related to the smaller size of this lesions in cirrhosis as compared to non-cirrhotic liver and to the heterogeneous fibrotic changes of the cirrhotic liver parenchyma.

### Hepatocellular carcinoma

Approximately 80–90% of cases of HCCs develop in cirrhotic patients [9].

**Fig. 12:**
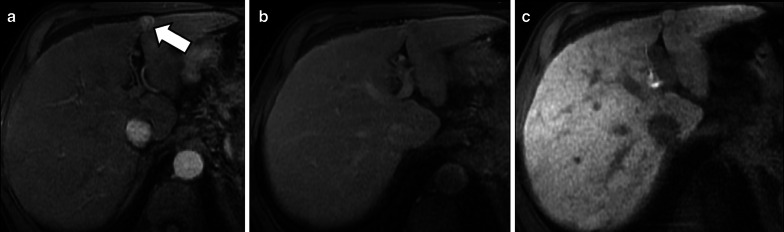
A 65-year-old patient with HCV-related cirrhosis and hepatocellular carcinoma. Gadobenate dimeglumine-enhanced MRI shows an HCC with (**a**) arterial phase hyperenhancement (arrow) in the arterial phase, (**b**) non-peripheral washout in the portal venous phase and (**c**) iso-hyperintensity in the hepatobiliary phase surrounded by a non-enhancing capsule

**Fig. 13:**
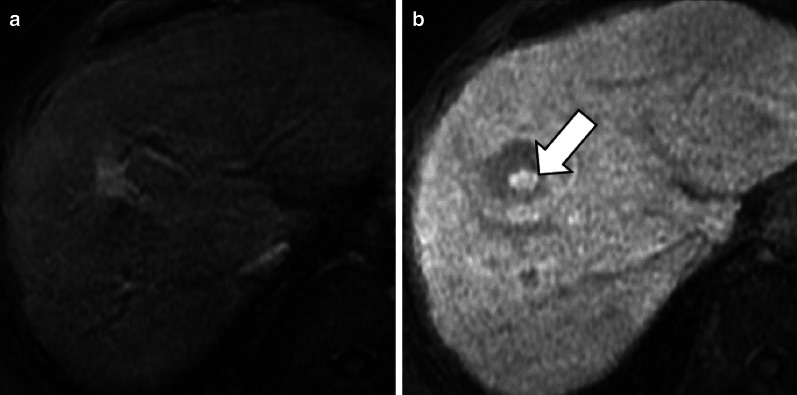
A 72-year-old man with HCV-related cirrhosis. Gadoxetate disodium-enhanced MRI shows a lesion with (**a**) arterial phase hyperenhancement and (**b**) nodule-in-nodule architecture with a smaller inner hyperintense nodule (arrow) within a larger outer hypointense nodule in hepatobiliary phase

As most HCCs show hypointensity on HBP, the Liver Imaging Reporting And Data System (LI-RADS) considers hypointensity on HBP an ancillary feature suggesting malignancy and isointensity on HBP an ancillary feature suggesting benignity [78]. However, HCCs show contrast uptake on HBP in 8.8–14% of the cases [76, 79]. A study by Asayama et al. [80] has reported an unexpectedly higher rate of uptake (Fig. 12), but this may be attributed to the different definition of uptake on HBP in this study (i.e., increase in signal intensity of the lesion on HBP compared with the precontrast image). Among the HCCs showing hyperintensity on HBP, the pattern of hyperintensity may be homogeneous, mosaic or as nodule-in-nodule in 57%, 29% and 14% of the cases, respectively [38]. Particularly, the nodule-in-nodule hyperintensity (Fig. 13) has been reported as a marker of the hepatocarcinogenesis process that can predate the appearance of hyperenhancement in hypovascular hypointense nodules [81]. This uptake is correlated with maintenance of hepatocyte function with upregulation of OATP1B3 and HNF 4α expression [82–84]. Prior studies [85–87] suggested that an abnormality in the expression or site of MRPs in the hepatocytes may correlate with hyperintensity on HBP, but this theory is still controversial.

**Fig. 14:**
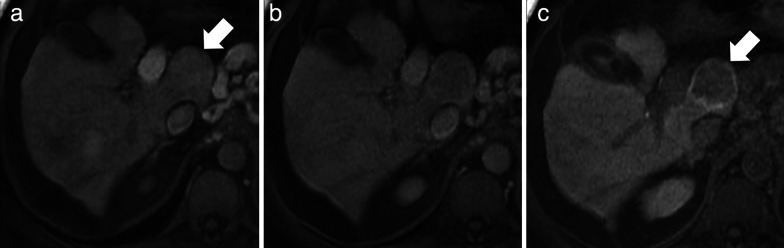
A 55-year-old man with HCV-related cirrhosis and multiple HCCs. Gadoxetate disodium-enhanced MRI shows an HCC mass in the caudate lobe with (**a**) arterial phase hyperenhancement in the arterial phase, (**b**) non-peripheral washout in the portal venous phase and (**c**) hypointensity in the hepatobiliary phase with peripheral hyperintensity (arrow), suggesting microvascular invasion

Recently, Yoneda et al. [88] described a pattern of HCC hypointense on HBP showing peritumoral hyperintensity (Fig. 14). This peritumoral hyperintensity on HBP may occur in HCC and may surround partially or completely the lesion, indicating the presence of peritumoral hyperplasia with glutamine synthetase and OATP1B3 expression [88]. The clinical relevance of the peritumoral hyperintensity on HBP is the higher incidence of microscopic hepatic venous invasion when this finding is detected [88].

### Regenerative and dysplastic nodules

Cirrhosis-associated regenerative nodules are innumerable well-defined nodules scattered within cirrhotic parenchyma, surrounded by scar tissue and typically measuring 1–15 mm in diameter [26].

**Fig. 15:**
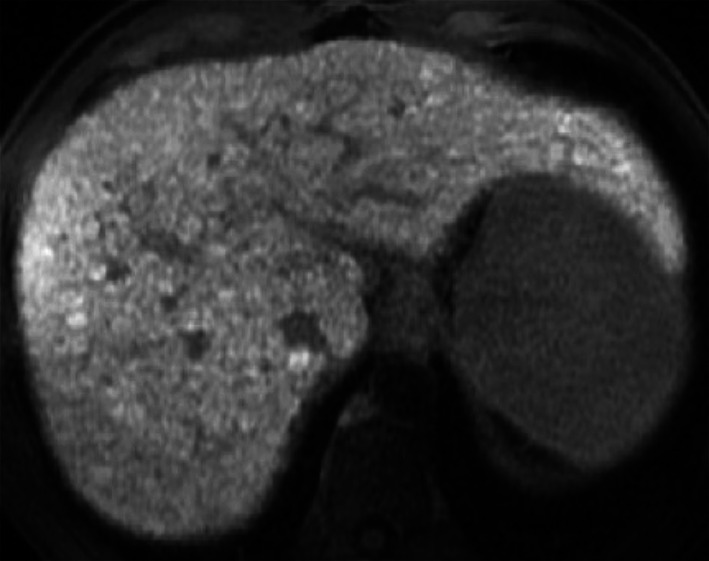
A 43-year-old man with HCV-related cirrhosis and multiple cirrhotic regenerative nodules. Gadoxetate disodium-enhanced MRI shows multiple cirrhotic regenerative nodules that are hyperintense on hepatobiliary phase

Regenerative nodules are well-defined regions of parenchyma made of hyperplastic hepatocytes that often contain ductular proliferation and are a response to necrosis, altered circulation or other stimuli. They may contain one (monoacinar) or multiple (multiacinar) portal tracts. Monoacinar nodules are usually 0.1–10 mm in diameter, while large multiacinar nodules are usually 5–15 mm in diameter [26]. These nodules show similar uptake of gadoxetate disodium to the surrounding liver tissue and thus appear isointense. However occasionally they may appear hyperintense when compared to the background tissue. Although the exact mechanism is still unknown, possible explanations include overexpression of OATP1B3 or down-regulation of MRP3 (Fig. 15) [26].

Dysplastic nodules are observed in up to 25% of cirrhotic patients [89]. Low- and high-grade dysplastic nodules commonly show iso- or hyperintensity relative to the surrounding liver in the HBP due to preserved OATP1B3 expression, but one-third of high-grade dysplastic nodules may be mildly hypointense [76].

### Multiacinar regenerative nodules

**Fig. 16:**
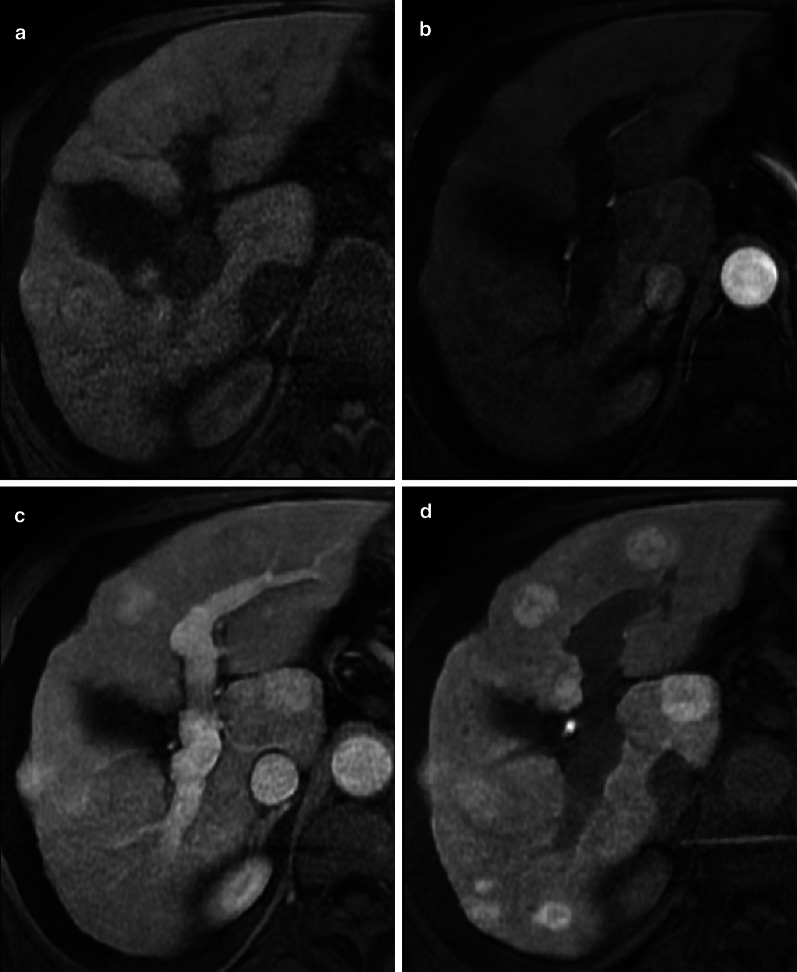
A 71-year-old man with HCV-related cirrhosis and multiacinar cirrhotic nodules. Gadoxetate disodium-enhanced MR shows multiple multiacinar cirrhotic nodules that are (**a**) isointense to surrounding liver parenchyma in the precontrast T1-weighted sequence and (**b**) in the hepatic arterial phase, (**c**) show enhancement in the portal venous phase due to early uptake of hepatobiliary contrast and (**d**) are hyperintense in the hepatobiliary phase

Multiacinar regenerative nodules with hyperintense rim on HBP develop in about 6% of cirrhotic patients, being more common in HBV-related cirrhosis than in HCV-related cirrhosis [80]. These lesions are usually multiple, do not show any arterial phase hyperenhancement, and may demonstrate hyperintensity on T2-weighted and diffusion-weighted images in 36% and 24% of the cases, respectively, mainly in the central area. These nodules may show an hyperintense rim on HBP with a doughnut-like appearance (Fig. 16) [90]. The reason of hyperintensity in the HBP compared to the surrounding regenerative nodules may be probably due to more hyperplastic change than surrounding monoacinar cirrhotic nodules [90]. At pathology, these lesions may correspond to multiacinar cirrhotic nodules in the International Working Party classification [26, 90]. Considering the lack of malignant potential of multiacinar regenerative nodules, these lesions do not require further investigations and can be managed conservatively [90].

## Periportal HBP hyperintensity

**Fig. 17:**
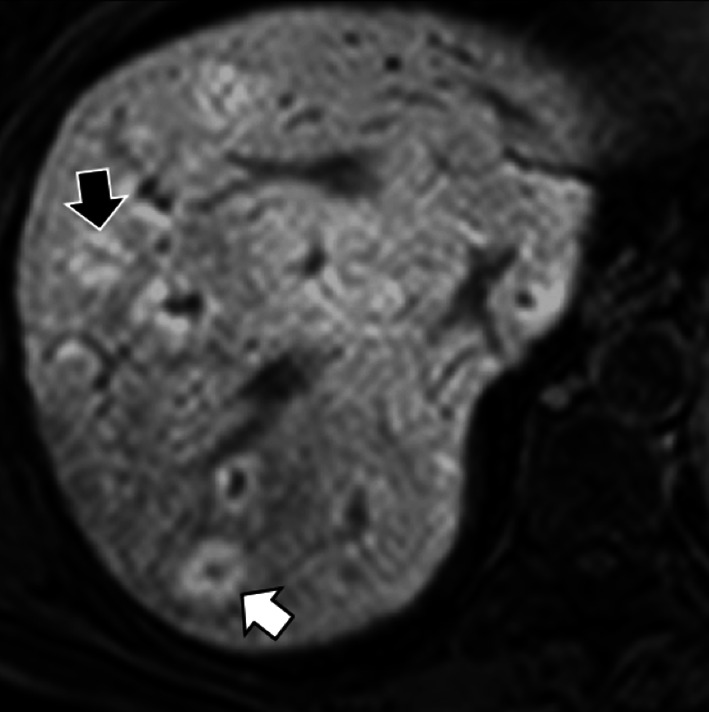
A 57-year-old woman with secondary sclerosing cholangitis and periportal hyperintensity on hepatobiliary phase. Gadoxetate disodium-enhanced MRI shows both thick tramline-like periportal hyperintensity (black arrow) and nodular-like periportal hyperintensity (white arrow)

Patients with various hepatobiliary diseases (e.g., liver cirrhosis, autoimmune hepatitis, primary sclerosing cholangitis, primary biliary cirrhosis and idiopathic portal hypertension) may show periportal hyperintensity in the HBP in 3% of cases (Fig. 17) [91]. Periportal hyperintensity in the HBP is defined as relatively higher enhancement bandlike areas along the portal tracts which take the form of a periportal ring or tramline and lower enhancement of the remaining areas of the liver in the HBP [91, 92]. The width of the hyperintense part may vary from less than 2 mm in the mild pattern to more than 3 mm in the severe pattern [88], and periportal HBP hyperintensity corresponds to periportal hyperintensity on T2-weighted images in 37% of the cases [92].

The causative mechanism and the clinical relevance of this imaging finding are still unclear. In patients with hepatic disorders such as primary biliary cirrhosis and idiopathic portal hypertension, it has been suggested that the periportal HBP hyperintensity is related to regenerative changes of periportal hepatocytes, which lead to a relatively increased uptake of the hepatobiliary contrast agent compared to the damaged background liver [91]. Onishi et al. [82] have also suggested that in patients with periportal hyperintensity in both HBP and T2-weighted images, the imaging finding indicates periportal edema which reflects the layer of loose connective tissue surrounding the portal veins expanded by inflammation or other conditions; consequently, periportal HBP hyperintensity could be considered as delayed enhancement of the periportal loose connective tissue in these patients.

## Practical tips based on the clinical setting

### Choice of the contrast agent based on clinical setting

In current practice, the use of extracellular contrast agents usually allows to determine the diagnosis of most focal liver lesions and should be favored as first imaging approach for the characterization of focal liver lesions and as baseline and follow-up imaging in oncologic patients. When the lesion is deemed indeterminate in studies with extracellular agents, the adoption of hepatobiliary MRI contrast agents is particularly relevant for the differential diagnosis between FNH and hepatocellular adenoma in the non-cirrhotic liver [11, 34–36] and between FNH-like nodules and HCC or metastases in vascular liver diseases and oncologic patients, respectively [22, 23, 53, 57–59, 67, 68]. In oncologic patients, hepatobiliary MRI contrast agents increase sensitivity for the detection of metastases as compared to extracellular agents [67, 68], and this is particularly relevant in patients with hepatic steatosis following chemotherapy [93] or for a complete staging in patients with colon cancer that are indicated surgery to uncover small liver metastases prior to surgery. Finally, in cirrhotic patients with prior history of HCC, HBP images are helpful to identify the loss of OATP8 expression in hypervascular lesions lacking washout to identify their progression toward malignancy [75–78] and to differentiate between malignancy and other benign entities such as regenerative nodules or multiacinar regenerative nodules that in some cases may pose diagnostic challenges.

### Diagnostic algorithm

When a focal liver observation shows iso- or hyperintensity in the HBP, our imaging evaluation should consider the clinical setting, the pattern of iso- or hyperintensity in the HBP and the information provided by extracellular images and T1-, T2-, and diffusion-weighted images.

A nodule showing a doughnut-like hyperintense pattern in the HBP usually indicates FNH in healthy patients [10, 11, 24, 32–39], FNH-like nodules in patients with vascular disease [50–54] or in oncologic patients after oxaliplatin therapy [56, 57] or multiacinar regenerative nodules in cirrhotic patients [90].

In case of a nodule showing central uptake of contrast agent in the HBP due to fibrotic content, imaging assessment should be based on extracellular phases: If the lesion shows irregular peripheral enhancement in the hepatic arterial phase and gradual centripetal enhancement on following phases, the diagnosis of intrahepatic cholangiocarcinoma is favored because this entity may show central uptake in 42–57% of cases [18–20]; if the patient has a history of malignancy and a target rim appearance on post-contrast phases, the lesion is suspicious for metastasis although central uptake in the HBP is not a common imaging presentation of liver metastases [22, 23, 67].

In case of iso- or hyperintense nodules on HBP lacking a doughnut-like pattern or central uptake, our diagnostic approach should be based on the following three scenarios:

1. if the lesion shows lack of signal drop on opposed phase compared to in-phase images in a steatotic liver and is not visible on T2- and T1- and diffusion-weighted images and extracellular phase, the diagnosis of fat sparing in steatotic liver is favored [17, 48];
2. in healthy or oncologic patients, if the lesion is highly hypervascular on arterial phase, and nearly isointense to liver parenchyma on T2-, T1- and diffusion-weighted images and extracellular phase, the diagnosis of FNH or FNH-like lesion should be favored, respectively;
3. in cirrhotic patients, our imaging evaluation should be aimed at excluding the presence of the small proportion of HCC that may show hyperintensity in the HBP; therefore, radiologists should first analyze extracellular phases, then should assess if the lesion contains intracellular fat on dual phase images and intensity on T1-, T2- and diffusion-weighted images. In case of lack of worrisome features for HCC (e.g., arterial phase hyperenhancement and washout on portal venous or delayed washout, fat content within lesion, hyperintensity on T2-weighted images or diffusion restriction) and the presence of hyperintensity on pre-contrast T1-weighted images, and if the lesion is less than 1 cm, the hyperintensity on HBP may indicate the presence of regenerative or low-grade dysplastic nodules.

In addition to the above considerations and prior to any decision on patient management, it is important to investigate whether the patient has any prior cross-sectional imaging available and to compare all prior examinations, particularly the oldest available one, with the current examination, in order to assess for lesion stability in size or changes of imaging presentations over time.

If the lesion showing iso- or hyperintensity on HBP is suspicious for hepatocellular adenomas, biopsy should be indicated to assess if the lesion has the β-catenin mutation because β-catenin hepatocellular adenomas are indicated to surgery due to their risk of malignant transformation [27, 43–45]. If the lesion is suspicious for malignancy (i.e., HCC, cholangiocarcinoma or metastases) but without a definitive imaging diagnosis, biopsy should be indicated to allow for a better patient-tailored management.

In conclusion, the presence of hyperintensity on HBP may be useful for the diagnosis of numerous benign and malignant hepatic masses based on knowledge of the clinical setting. Indeed, FNH and FNH-like lesions are likely the most common lesions showing hyperintensity on HBP in patients without cirrhosis. In cirrhotic and in oncologic patients, well-differentiated HCC are usually hypointense on HBP but may show hyperintensity in the HBP in about 9–14% of cases, while cholangiocarcinoma and some metastases may demonstrate variable inner signal characteristics with a peripheral rim of hypointensity. In such scenarios, malignancy must be ruled out before considering the diagnosis of a benign condition, and the use of hepatobiliary MRI contrast agents proves to be particularly useful. Finally, a histopathological examination may be required to resolve challenging cases.

## Data Availability

The datasets used and/or analyses during the current study are available from the corresponding author on reasonable request.

## Abbreviations

FNH: Focal nodular hyperplasia
HBP: Hepatobiliary phase
HCAs: Hepatocellular adenomas
HCC: Hepatocellular carcinoma
MRP2: Multidrug-resistance-associated proteins
OATP1B3: Organic anion transporting polypeptide 1

## References

[1] Van Beers BE, Pastor CM, Hussain HK (2012). Primovist, Eovist: what to expect?. J Hepatol.

[2] Dahlqvist Leinhard O, Dahlström N, Kihlberg J (2012). Quantifying differences in hepatic uptake of the liver specific contrast agents Gd-EOB-DTPA and Gd-BOPTA: a pilot study. Eur Radiol.

[3] Spinazzi A, Lorusso V, Pirovano G, Kirchin M (1999). Safety, tolerance, biodistribution, and MR imaging enhancement of the liver with gadobenate dimeglumine: results of clinical pharmacologic and pilot imaging studies in nonpatient and patient volunteers. Acad Radiol.

[4] Brismar TB, Dahlstrom N, Edsborg N, Persson A, Smedby O, Albiin N (2009). Liver vessel enhancement by Gd-BOPTA and Gd-EOB-DTPA: a comparison in healthy volunteers. Acta Radiol.

[5] Feuerlein S, Gupta RT, Boll DT, Merkle EM (2012). Hepatocellular MR contrast agents: enhancement characteristics of liver parenchyma and portal vein after administration of gadoxetic acid in comparison to gadobenate dimeglumine. Eur J Radiol.

[6] Vernuccio F, Cannella R, Gozzo C (2020). Hepatic enhancement in cirrhosis in the portal venous phase: what are the differences between gadoxetate disodium and gadobenate dimeglumine?. Abdom Radiol (NY).

[7] Yoneda N, Matsui O, Kitao A (2016). Benign hepatocellular nodules: hepatobiliary phase of gadoxetic acid-enhanced MR imaging based on molecular background. Radiographics.

[8] Campos JT, Sirlin CB, Choi JY (2012). Focal hepatic lesions in Gd-EOB-DTPA enhanced MRI: the atlas. Insights Imaging.

[9] Hansen N, Weadock W, Morani A (2012). Liver lesions discovered incidentally on ultrasound: evaluation of reader ability to characterize lesions on MRI without intravenous contrast. Acad Radiol.

[10] Suh CH, Kim KW, Kim GY, Shin YM, Kim PN, Park SH (2015). The diagnostic value of Gd-EOB-DTPA-MRI for the diagnosis of focal nodular hyperplasia: a systematic review and meta-analysis. Eur Radiol.

[11] McInnes MD, Hibbert RM, Inácio JR, Schieda N (2015). Focal nodular hyperplasia and hepatocellular adenoma: accuracy of gadoxetic acid-enhanced MR imaging—a systematic review. Radiology.

[12] Reddy SK, Kishnani PS, Sullivan JA (2007). Resection of hepatocellular adenoma in patients with glycogen storage disease type Ia. J Hepatol.

[13] Thomeer MG, Willemssen FE, Biermann KK (2014). MRI features of inflammatory hepatocellular adenomas on hepatocyte phase imaging with liver-specific contrast agents. J Magn Reson Imaging.

[14] Glockner JF, Lee CU (2017). Mounajjed T inflammatory hepatic adenomas: characterization with hepatobiliary MRI contrast agents. Magn Reson Imaging..

[15] Auer TA, Fehrenbach U, Grieser C (2020). Hepatocellular adenomas: is there additional value in using Gd-EOB-enhanced MRI for subtype differentiation?. Eur Radiol.

[16] Denecke T, Steffen IG, Agarwal S (2012). Appearance of hepatocellular adenomas on gadoxetic acid-enhanced MRI. Eur Radiol.

[17] Ünal E, İdilman İS, Karaosmanoğlu AD (2019). Hyperintensity at fat spared area in steatotic liver on the hepatobiliary phase MRI. Diagn Interv Radiol.

[18] Jeong HT, Kim MJ, Chung YE, Choi JY, Park YN, Kim KW (2013). Gadoxetate disodium-enhanced MRI of mass-forming intrahepatic cholangiocarcinomas: imaging-histologic correlation. AJR Am J Roentgenol.

[19] Koh J, Chung YE, Nahm JH (2016). Intrahepatic mass-forming cholangiocarcinoma: prognostic value of preoperative gadoxetic acid-enhanced MRI. Eur Radiol.

[20] Min JH, Kim YK, Choi SY (2017). Differentiation between cholangiocarcinoma and hepatocellular carcinoma with target sign on diffusion-weighted imaging and hepatobiliary phase gadoxetic acid-enhanced MR imaging: Classification tree analysis applying capsule and septum. Eur J Radiol.

[21] Park HJ, Kim YK, Park MJ, Lee WJ (2013). Small intrahepatic mass-forming cholangiocarcinoma: target sign on diffusion-weighted imaging for differentiation from hepatocellular carcinoma. Abdom Imaging.

[22] Ha S, Lee CH, Kim BH (2012). Paradoxical uptake of Gd-EOB-DTPA on the hepatobiliary phase in the evaluation of hepatic metastasis from breast cancer: is the “target sign” a common finding?. Magn Reson Imaging.

[23] Kim A, Lee CH, Kim BH (2012). Gadoxetic acid-enhanced 3.0T MRI for the evaluation of hepatic metastasis from colorectal cancer: metastasis is not always seen as a “defect” on the hepatobiliary phase. Eur J Radiol.

[24] Nguyen BN, Fléjou JF, Terris B (1999). Focal nodular hyperplasia of the liver: a comprehensive pathologic study of 305 lesions and recognition of new histologic forms. Am J Surg Pathol.

[25] Kaltenbach TE, Engler P, Kratzer W (2016). Prevalence of benign focal liver lesions: ultrasound investigation of 45,319 hospital patients. Abdom Radiol (NY).

[26] International Working Party (1995). Terminology of nodular hepatocellular lesions. Hepatology.

[27] European Association for the Study of the Liver (EASL) (2016). EASL clinical practice guidelines on the management of benign liver tumours. J Hepatol.

[28] Vernuccio F, Ronot M, Dioguardi Burgio M (2018). Uncommon evolutions and complications of common benign liver lesions. Abdom Radiol (NY).

[29] Yoneda N, Matsui O, Kitao A (2012). Hepatocyte transporter expression in FNH and FNH-like nodule: correlation with signal intensity on gadoxetic acid enhanced magnetic resonance images. Jpn J Radiol.

[30] Fujiwara H, Sekine S, Onaya H (2011). Ring-like enhancement of focal nodular hyperplasia with hepatobiliary-phase Gd-EOB-DTPA-enhanced magnetic resonance imaging: radiological-pathological correlation. Jpn J Radiol.

[31] Bioulac-Sage P, Cubel G, Taouji S (2012). Immunohistochemical markers on needle biopsies are helpful for the diagnosis of focal nodular hyperplasia and hepatocellular adenoma subtypes. Am J Surg Pathol.

[32] Reizine E, Amaddeo G, Pigneur F (2018). Quantitative correlation between uptake of Gd-BOPTA on hepatobiliary phase and tumor molecular features in patients with benign hepatocellular lesions. Eur Radiol.

[33] van Kessel CS, de Boer E, ten Kate FJ, Brosens LA, Veldhuis WB, van Leeuwen MS (2013). Focal nodular hyperplasia: hepatobiliary enhancement patterns on gadoxetic-acid contrast-enhanced MRI. Abdom Imaging.

[34] Bieze M, van den Esschert JW, Nio CY (2012). Diagnostic accuracy of MRI in differentiating hepatocellular adenoma from focal nodular hyperplasia: prospective study of the additional value of gadoxetate disodium. AJR Am J Roentgenol.

[35] Grieser C, Steffen IG, Kramme IB (2014). Gadoxetic acid enhanced MRI for differentiation of FNH and HCA: a single centre experience. Eur Radiol.

[36] An HS, Park HS, Kim YJ, Jung SI, Jeon HJ (2013). Focal nodular hyperplasia: characterisation at gadoxetic acid-enhanced MRI and diffusion-weighted MRI. Br J Radiol.

[37] Mohajer K, Frydrychowicz A, Robbins JB, Loeffler AG, Reed TD, Reeder SB (2012). Characterization of hepatic adenoma and focal nodular hyperplasia with gadoxetic acid. J Magn Reson Imaging.

[38] Kitao A, Matsui O, Yoneda N (2018). Differentiation between hepatocellular carcinoma showing hyperintensity on the hepatobiliary phase of gadoxetic acid-enhanced MRI and focal nodular hyperplasia by CT and MRI. AJR Am J Roentgenol.

[39] Grazioli L, Bondioni MP, Haradome H (2012). Hepatocellular adenoma and focal nodular hyperplasia: value of gadoxetic acid-enhanced MR imaging in differential diagnosis. Radiology.

[40] Giovanoli O, Heim M, Terracciano L (2008). MRI of hepatic adenomatosis: initial observations with gadoxetic acid contrast agent in three patients. AJR Am J Roentgenol.

[41] Gevers TJG, Marcel Spanier BW, Veendrick PB, Vrolijk JM (2018). Regression of hepatocellular adenoma after bariatric surgery in severe obese patients. Liver Int.

[42] Vernuccio F, Ronot M, Dioguardi Burgio M (2020). Long-term evolution of hepatocellular adenomas at MRI follow-up. Radiology.

[43] Nault JC, Couchy G, Balabaud C (2017). Molecular classification of hepatocellular adenoma associates with risk factors, bleeding, and malignant transformation. Gastroenterology.

[44] Ba-Ssalamah A, Antunes C, Feier D (2015). Morphologic and molecular features of hepatocellular adenoma with gadoxetic acid-enhanced MR imaging. Radiology.

[45] Sciarra A, Schmidt S, Pellegrinelli A (2019). OATPB1/B3 and MRP3 expression in hepatocellular adenoma predicts Gd-EOB-DTPA uptake and correlates with risk of malignancy. Liver Int.

[46] Yoneda N, Matsui O, Kitao A (2012). Beta-catenin-activated hepatocellular adenoma showing hyperintensity on hepatobiliary-phase gadoxetic-enhanced magnetic resonance imaging and overexpression of OATP8. Jpn J Radiol.

[47] Agarwal S, Fuentes-Orrego JM, Arnason T (2014). Inflammatory hepatocellular adenomas can mimic focal nodular hyperplasia on gadoxetic acid-enhanced MRI. AJR Am J Roentgenol.

[48] Dioguardi Burgio M, Bruno O, Agnello F (2016). The cheating liver: imaging of focal steatosis and fatty sparing. Expert Rev Gastroenterol Hepatol.

[49] Vilgrain V, Paradis V, Van Wettere M (2018). Benign and malignant hepatocellular lesions in patients with vascular liver diseases. Abdom Radiol (NY).

[50] Marin D, Galluzzo A, Plessier A, Brancatelli G, Valla D, Vilgrain V (2011). Focal nodular hyperplasia-like lesions in patients with cavernous transformation of the portal vein: prevalence, MR findings and natural history. Eur Radiol.

[51] Baiges A, Turon F, Simón-Talero M (2020). Congenital extrahepatic portosystemic shunts (abernethy malformation): an international observational study. Hepatology.

[52] Brenard R, Chapaux X, Deltenre P (2010). Large spectrum of liver vascular lesions including high prevalence of focal nodular hyperplasia in patients with hereditary haemorrhagic telangiectasia: the Belgian Registry based on 30 patients. Eur J Gastroenterol Hepatol.

[53] Vilgrain V, Lewin M, Vons C (1999). Hepatic nodules in Budd–Chiari syndrome: imaging features. Radiology.

[54] Lee YH, Kim SH, Cho MY, Shim KY, Kim MS (2007). Focal nodular hyperplasia-like nodules in alcoholic liver cirrhosis: radiologic-pathologic correlation. AJR Am J Roentgenol.

[55] Galia M, Taibbi A, Marin D (2014). Focal lesions in cirrhotic liver: what else beyond hepatocellular carcinoma?. Diagn Interv Radiol.

[56] Furlan A, Brancatelli G, Dioguardi Burgio M (2018). Focal nodular hyperplasia after treatment with oxaliplatin: a multiinstitutional series of cases diagnosed at MRI. AJR Am J Roentgenol.

[57] Rubbia-Brandt L, Lauwers GY, Wang H (2010). Sinusoidal obstruction syndrome and nodular regenerative hyperplasia are frequent oxaliplatin-associated liver lesions and partially prevented by bevacizumab in patients with hepatic colorectal metastasis. Histopathology.

[58] Yang HK, Jang HJ, Khalili K, Wald RM, Yoo SJ, Kim TK (2020). CT and MR imaging findings of the livers in adults with Fontan palliation: an observational study. Abdom Radiol (NY).

[59] Mamone G, Carollo V, Di Piazza A, Cortis K, Degiorgio S, Miraglia R (2019). Budd–Chiari syndrome and hepatic regenerative nodules: magnetic resonance findings with emphasis of hepatobiliary phase. Eur J Radiol.

[60] Bruneton JN, Raffaelli C, Maestro C (1995). Benign liver lesions: implications of detection in cancer patients. Eur Radiol.

[61] Schwartz LH, Gandras EJ, Colangelo S (1999). Prevalence and importance of small hepatic lesions found at CT in patients with cancer. Radiology.

[62] Jones EC, Chezmar JL, Nelson RC, Bernardino ME (1992). The frequency and significance of small (less than or equal to 15 mm) hepatic lesions detected by CT. AJR Am J Roentgenol.

[63] Jang HJ, Lim HK, Lee WJ, Lee SJ, Yun JY, Choi D (2002). Small hypoattenuating lesions in the liver on single-phase helical CT in preoperative patients with gastric and colorectal cancer: prevalence, significance, and differentiating features. J Comput Assist Tomogr.

[64] Expert Panel on Gastrointestinal Imaging (2017). ACR Appropriateness Criteria® suspected liver metastases. J Am Coll Radiol.

[65] de Ridder J, de Wilt JH, Simmer F (2016). Incidence and origin of histologically confirmed liver metastases: an explorative case-study of 23,154 patients. Oncotarget.

[66] Jeong HT, Kim MJ, Park MS (2012). Detection of liver metastases using gadoxetic-enhanced dynamic and 10- and 20-minute delayed phase MR imaging. J Magn Reson Imaging.

[67] Granata V, Fusco R, de Lutio di Castelguidone E (2019). Diagnostic performance of gadoxetic acid-enhanced liver MRI versus multidetector CT in the assessment of colorectal liver metastases compared to hepatic resection. BMC Gastroenterol.

[68] Kim YK, Lee MW, Lee WJ (2012). Diagnostic accuracy and sensitivity of diffusion-weighted and of gadoxetic acid-enhanced 3-T MR imaging alone or in combination in the detection of small liver metastasis (≤ 1.5 cm in diameter). Invest Radiol.

[69] Park SH, Kim H, Kim EK (2017). Aberrant expression of OATP1B3 in colorectal cancer liver metastases and its clinical implication on gadoxetic acid-enhanced MRI. Oncotarget.

[70] Wlcek K, Svoboda M, Riha J (2011). The analysis of organic anion transporting polypeptide (OATP) mRNA and protein patterns in primary and metastatic liver cancer. Cancer Biol Ther.

[71] Mahfouz AE, Hamm B, Wolf KJ (1994). Peripheral washout: a sign of malignancy on dynamic gadolinium-enhanced MR images of focal liver lesions. Radiology.

[72] Khan SA, Thomas HC, Davidson BR (2005). Cholangiocarcinoma. Lancet.

[73] Kang Y, Lee JM, Kim SH, Han JK, Choi BI (2012). Intrahepatic mass-forming cholangiocarcinoma: enhancement patterns on gadoxetic acid-enhanced MR images. Radiology.

[74] Jeon TY, Kim SH, Lee WJ (2010). The value of gadobenate dimeglumine-enhanced hepatobiliary-phase MR imaging for the differentiation of scirrhous hepatocellular carcinoma and cholangiocarcinoma with or without hepatocellular carcinoma. Abdom Imaging.

[75] Vernuccio F, Cannella R, Meyer M (2019). LI-RADS: diagnostic performance of hepatobiliary phase hypointensity and major imaging features of LR-3 and LR-4 lesions measuring 10–19 mm with arterial phase hyperenhancement. AJR Am J Roentgenol.

[76] Kitao A, Matsui O, Yoneda N (2011). The uptake transporter OATP8 expression decreases during multistep hepatocarcinogenesis: correlation with gadoxetic acid enhanced MR imaging. Eur Radiol.

[77] Liu X, Zou L, Liu F, Zhou Y, Song B (2013). Gadoxetic acid disodium-enhanced magnetic resonance imaging for the detection of hepatocellular carcinoma: a meta-analysis. PLoS ONE.

[78] https://www.acr.org/Clinical-Resources/Reporting-and-Data-Systems/LI-RADS/CT-MRI-LI-RADS-v2018. Accessed 16 Sept 2020.

[79] Kim JY, Kim MJ, Kim KA, Jeong HT, Park YN (2012). Hyperintense HCC on hepatobiliary phase images of gadoxetic acid-enhanced MRI: correlation with clinical and pathological features. Eur J Radiol.

[80] Asayama Y, Tajima T, Nishie A (2011). Uptake of Gd-EOB-DTPA by hepatocellular carcinoma: radiologic-pathologic correlation with special reference to bile production. Eur J Radiol.

[81] Cannella R, Calandra A, Cabibbo G (2019). Hyperintense nodule-in-nodule on hepatobiliary phase arising within hypovascular hypointense nodule: outcome and rate of hypervascular transformation. Eur J Radiol.

[82] Yamashita T, Kitao A, Matsui O (2014). Gd-EOB-DTPA-enhanced magnetic resonance imaging and alpha-fetoprotein predict prognosis of early-stage hepatocellular carcinoma. Hepatology.

[83] Narita M, Hatano E, Arizono S (2009). Expression of OATP1B3 determines uptake of Gd-EOB-DTPA in hepatocellular carcinoma. J Gastroenterol.

[84] Kitao A, Matsui O, Yoneda N (2020). Gadoxetic acid-enhanced MR imaging for hepatocellular carcinoma: molecular and genetic background. Eur Radiol.

[85] Tsuboyama T, Onishi H, Kim T (2010). Hepatocellular carcinoma: hepatocyte-selective enhancement at gadoxetic acid-enhanced MR imaging—correlation with expression of sinusoidal and canalicular transporters and bile accumulation. Radiology.

[86] Ueno A, Masugi Y, Yamazaki K (2014). OATP1B3 expression is strongly associated with Wnt/beta-catenin signalling and represents the transporter of gadoxetic acid in hepatocellular carcinoma. J Hepatol.

[87] Kitao A, Zen Y, Matsui O (2010). Hepatocellular carcinoma: signal intensity at gadoxetic acid-enhanced MR imaging—correlation with molecular transporters and histopathologic features. Radiology.

[88] Yoneda N, Matsui O, Kitao A (2018). Peri-tumoral hyperintensity in the hepatobiliary phase of gadoxetic acid-enhanced MRI in hepatocellular carcinomas: correlation with peri-tumoral hyperplasia and its pathological features. Abdom Radiol (NY).

[89] Theise ND (1996). Cirrhosis and hepatocellular neoplasia: more like cousins than like parent and child. Gastroenterology.

[90] Kozaka K, Kobayashi S, Yoneda N (2019). Doughnut-like hyperintense nodules in the hepatobiliary phase without arterial-phase hyperenhancement in cirrhotic liver: imaging and clinicopathological features. Eur Radiol.

[91] Kobayashi S, Matsui O, Gabata T (2013). Intrahepatic periportal high intensity on hepatobiliary phase images of Gd-EOB-DTPA-enhanced MRI: imaging findings and prevalence in various hepatobiliary diseases. Jpn J Radiol.

[92] Onishi H, Theisen D, Zachoval R, Reiser MF, Zech CJ (2019). Intrahepatic diffuse periportal enhancement patterns on hepatobiliary phase gadoxetate disodium-enhanced liver MR images: do they correspond to periportal hyperintense patterns on T2-weighted images?. Medicine (Baltimore).

[93] Vernuccio F, Dioguardi Burgio M, Barbiera F (2019). CT and MR imaging of chemotherapy-induced hepatopathy. Abdom Radiol (NY).

